# Host conditioning with IL-1β improves the antitumor function of adoptively transferred T cells

**DOI:** 10.1084/jem.20181218

**Published:** 2019-08-12

**Authors:** Ping-Hsien Lee, Tori N. Yamamoto, Devikala Gurusamy, Madhusudhanan Sukumar, Zhiya Yu, Jane Hu-Li, Takeshi Kawabe, Arunakumar Gangaplara, Rigel J. Kishton, Amanda N. Henning, Suman K. Vodnala, Ronald N. Germain, William E. Paul, Nicholas P. Restifo

**Affiliations:** 1 Surgery Branch, National Cancer Institute, National Institutes of Health, Bethesda, MD; 2 Center for Cell-Based Therapy, National Cancer Institute, National Institutes of Health, Bethesda, MD; 3 Cytokine Biology Unit, Laboratory of Immune System Biology, National Institute of Allergy and Infectious Diseases, National Institutes of Health, Bethesda, MD; 4 Lymphocyte Biology Section, Laboratory of Immune System Biology, National Institute of Allergy and Infectious Diseases, National Institutes of Health, Bethesda, MD; 5 Cellular Immunology Section, Laboratory of Immune System Biology, National Institute of Allergy and Infectious Diseases, National Institutes of Health, Bethesda, MD; 6 Immunology Graduate Group, University of Pennsylvania, Philadelphia, PA

## Abstract

Host conditioning has emerged as an important component of effective adoptive cell transfer–based immunotherapy for cancer. High levels of IL-1β are induced by host conditioning, but its impact on the antitumor function of T cells remains unclear. We found that the administration of IL-1β increased the population size and functionality of adoptively transferred T cells within the tumor. Most importantly, IL-1β enhanced the ability of tumor-specific T cells to trigger the regression of large, established B16 melanoma tumors in mice. Mechanistically, we showed that the increase in T cell numbers was associated with superior tissue homing and survival abilities and was largely mediated by IL-1β–stimulated host cells. In addition, IL-1β enhanced T cell functionality indirectly via its actions on radio-resistant host cells in an IL-2– and IL-15–dependent manner. Our findings not only underscore the potential of provoking inflammation to enhance antitumor immunity but also uncover novel host regulations of T cell responses.

## Abstract (precis)

Lee et al. reveal that administration of IL-1β can alter the host environment to augment the magnitude and functionality of CD8^+^ T cells, thereby improving the efficacy of adoptively transferred tumor-reactive T cells in treating large, established tumors in mice.

## Introduction

Adoptive transfer of antitumor T cells has shown great potential as an effective cancer immunotherapy. The infusion of tumor-infiltrating lymphocytes with inherent tumor reactivities or autologous T cells genetically modified to express tumor-reactive TCRs or chimeric antigen receptors can mediate durable tumor regression in several malignancies (Rosenberg et al., 2008; Rosenberg and Restifo, 2015; June et al., 2018; June and Sadelain, 2018). Although adoptive cell therapy (ACT)–based immunotherapy has made great strides forward in recent years, it remains ineffective for a majority of patients with common epithelial cancers (Tran et al., 2017).

Various efforts have focused on augmenting the expansion and function of adoptively transferred T cells, including the use of host preparative regimens such as nonmyeloablative chemotherapy and irradiation. Host lymphodepletion induced by nonmyeloablative conditioning enhances the efficacy of ACT through mechanisms that have not yet been fully elucidated but likely include a reduction of regulatory T cells (T reg cells), the elimination of cellular “sinks” for homeostatic cytokines such as IL-7 and IL-15, and the liberation of LPS from the gut microbiota (Antony et al., 2005; Gattinoni et al., 2005a; Paulos et al., 2007). Notably, high serum levels of IL-1β were found in parallel with LPS in mice that received total body irradiation (Paulos et al., 2007), possibly attributed to its high inducibility by LPS (Higgins et al., 1994). Given that IL-1β administration can enhance the protective value of vaccines (Ben-Sasson et al., 2013a,b; Wüthrich et al., 2013), we investigated its therapeutic potential in improving the efficacy of ACT for treating established tumors.

Herein we show that administration of IL-1β markedly improved the efficacy of adoptively transferred T cells in mediating tumor regression by increasing their cell numbers and functionality within the tumor. The cell number increase was associated with enhanced tissue trafficking and survival of T cells, and required IL-1R1 expression in both transferred T cells and host cells. By contrast, the enhanced functionality was not triggered directly by the IL-1R signaling pathway in T cells but relied on IL-1β–stimulated radio-resistant host cells in a TCR-independent manner. We further demonstrate that the augmented T cell functionality depended on IL-2 and IL-15. Dual blockade of IL-2 and IL-15 abrogated the IL-1β enhancement of ACT-mediated tumor regression. Collectively, our findings highlight the potent adjuvant activity of IL-1β in ACT for cancer treatment and delineate how inflammation shapes the host environment to modulate T cell responses.

## Results

### Administration of IL-1β enhances the antitumor function of adoptively transferred CD8^+^ T cells

**Figure 1.**
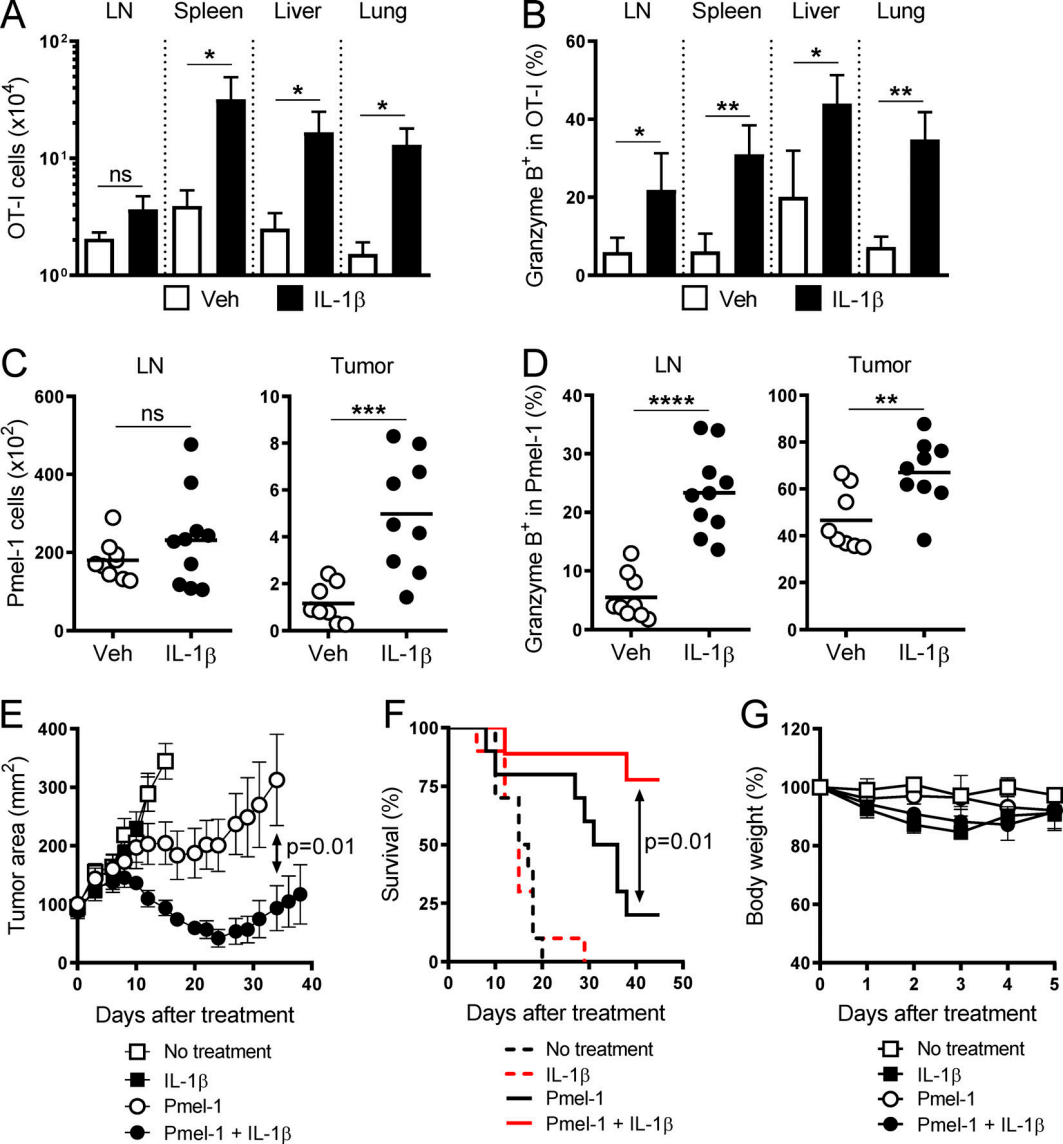
**Administration of IL-1β enhances the antitumor function of adoptively transferred CD8^+^ T cells. (A and B)** Absolute cell number and frequency of OT-I cells expressing Gzm B isolated on day 7 from vehicle (Veh; white bars)– and IL-1β (black bars)–treated mice as described in Fig. S1 A (*n* = 3). **(C and D)** Absolute cell number and frequency of Pmel-1 cells expressing Gzm B isolated on day 5 from vehicle (white circles)– and IL-1β (black circles)– treated mice as described in Fig. S1 D (*n* = 9 or 10). **(E–G)** Tumor area, overall survival, and changes in body weight of tumor-bearing mice as described in Fig. S1 D (*n* = 10 for E and F; *n* = 5 for G). Data are representative of five (A and B), two (C, D, and G), or four (E and F) independent experiments (*, P < 0.05; **, P < 0.01; ***, P < 0.001; ****, P < 0.0001; ns, not significant; error bars, SD for A, B, and G; SEM for E).

We have previously demonstrated that the systemic administration of IL-1β increased cell numbers and granzyme B (Gzm B) expression of adoptively transferred OT-I CD8^+^ T cells in both lymphoid and nonlymphoid tissues in response to OVA/LPS immunization (Ben-Sasson et al., 2013a). While IL-1β treatment in this context had a marked impact on OT-I cells, the infusion of high quantities of IL-1β (10 µg over 5 d) resulted in severe inflammation and subsequent animal morbidity and mortality. A regimen comprised of reduced dosage and shorter duration of IL-1β (6 µg over 4 d) was well tolerated by mice, and we evaluated its effects on OT-I cells (Fig. S1 A). This modified dosing strategy recapitulated the previously observed increases in OT-I cell numbers and Gzm B expression, with the exception of the cell number increase in the draining LNs (Fig. 1, A and B). Similar effects of IL-1β were also observed on endogenous OVA-specific SIINFEKL/H2-K^b^ tetramer^+^ CD8^+^ T cells (Fig. S1, B and C).

To verify these findings in another transgenic TCR model and to assess the impact of IL-1β on in vivo antitumor function, we used premelanosome protein-1 (Pmel-1) CD8^+^ T cells that recognize the H-2D^b^–restricted glycoprotein 100 (gp100)_25-33_ peptide (Overwijk et al., 2003). As described in Fig. S1 D, we inoculated mice with B16 melanoma tumor cells engineered to overexpress the altered gp100 peptide, hereinafter named B16-mhgp100 (Hanada et al., 2019), and allowed them to grow for 10 d. The mice bearing established B16-mhgp100 tumors then received total body irradiation, followed by the infusion of Pmel-1 cells and injections of vehicle or IL-1β. Tumor-draining LNs and tumor were harvested 1 d after the last injection. We found that IL-1β treatment increased Pmel-1 cell numbers only in the tumor but enhanced Gzm B expression in both the tumor-draining LNs and tumor (Fig. 1, C and D), consistent with the OT-I findings (Fig. 1, A and B).

Notably, even though IL-1β administration alone showed no impact on the growth of established B16-mhgp100 tumors or mouse survival, it improved the ability of adoptively transferred Pmel-1 cells to induce tumor regression and prolong mouse survival (Fig. 1, E and F). We also monitored the body weight of tumor-bearing mice during the treatment course and found that IL-1β only caused a transient weight loss regardless of the infusion of Pmel-1 cells (Fig. 1 G), and no mortality unrelated to tumor burden was observed. Together, these data suggest that IL-1β can be safely administered to improve the efficacy of ACT by enhancing the tumor infiltration and functionality of antitumor T cells.

### IL-1β endows CD8^+^ T cells with an effector-like gene signature

**Figure 2.**
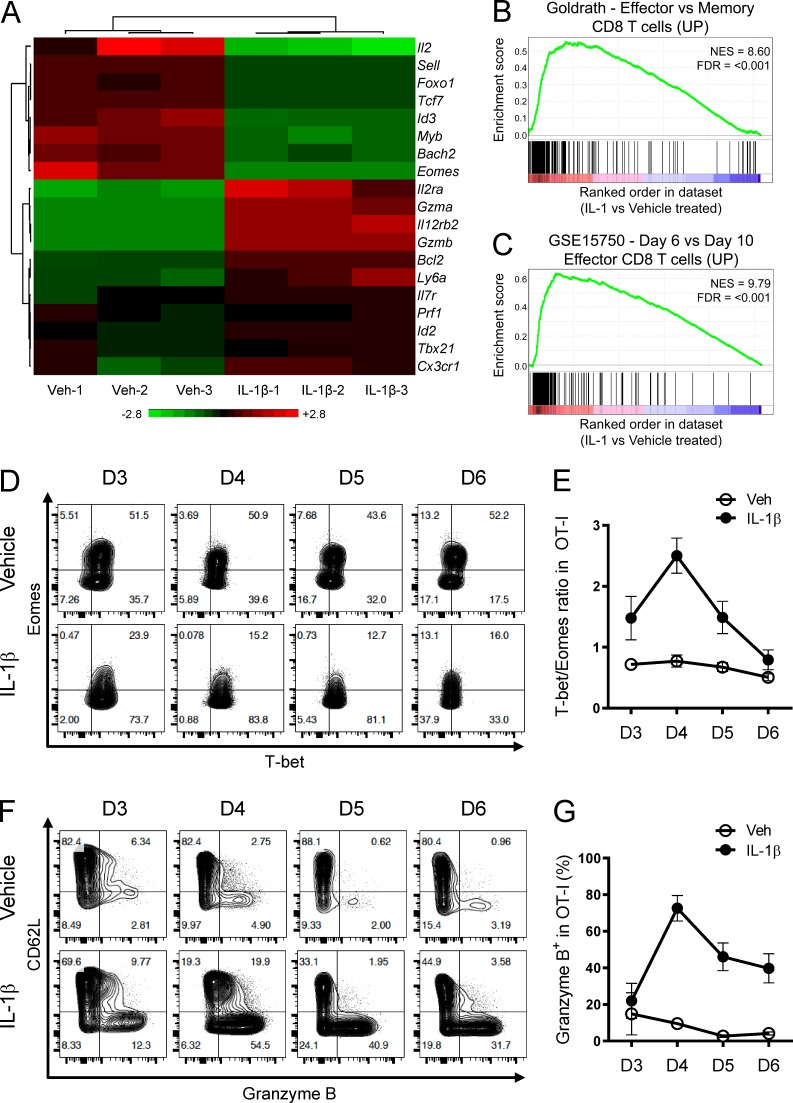
**IL-1β endows CD8^+^ T cells with an effector-like gene signature. (A)** RNA-seq analysis of vehicle- and IL-1β–exposed OT-I cells isolated from the draining LNs on day 4. Heatmap of selected genes associated with effector and memory differentiation is shown (*n* = 3). **(B and C)** GSEA plots showing the enrichment of effector-associated genes in IL-1β– versus vehicle-exposed OT-I cells as described in A. NES, normalized enrichment score; FDR, false discovery rate. **(D)** Representative contour plots showing T-bet and Eomes expression in vehicle- and IL-1β–exposed OT-I cells isolated from the draining LNs at indicated time points post OVA/LPS immunization. FTY720 was injected i.p. daily starting from day 2. Plots were gated on the live CD8^+^Vα2^+^CD45.1^+^CD45.2^–^ population. **(E)** Kinetics of T-bet/Eomes ratio as described in D (*n* = 3). **(F)** Representative contour plots showing Gzm B and CD62L expression as described in D. **(G)** Kinetics of Gzm B expression as described in D. **(D–G)** Representative of two independent experiments (error bars, SD).

To gain a better understanding of how IL-1β conferred the beneficial effects to T cells, we performed a genome-wide transcriptome analysis on OT-I cells isolated on day 4 from the draining LNs under the influence of vehicle or IL-1β (Fig. 2 A and Table S1). In addition to *Gzmb*, we found that several other effector-associated genes (such as *Gzma*, *Prf1*, *Il2ra*, and *Id2*) were up-regulated while memory-associated genes (such as *Il2*, *Sell*, *Foxo1*, *Tcf7*, and *Id3*) were down-regulated in IL-1β–exposed OT-I cells compared with vehicle-exposed counterparts. Gene set enrichment analysis (GSEA) also supports an effector-like profile, with genes up-regulated in IL-1β–exposed OT-I cells enriched for genes up-regulated in effector T cells generated during vaccinia virus (Luckey et al., 2006; Fig. 2 B) or *Listeria monocytogenes* infection (Pearce et al., 2009; Fig. 2 C).

To further interrogate *Gzmb* gene regulation at the molecular level, we focused on T-bet and eomesodermin (Eomes), two T-box transcription factors known to drive *Gzmb* expression directly and govern CD8^+^ T cell differentiation (Intlekofer et al., 2005; Lazarevic et al., 2013). Given that the balance between T-bet and Eomes can be fine-tuned by inflammatory cytokines to regulate effector cell differentiation (Takemoto et al., 2006; Joshi et al., 2007), we sought to determine if IL-1β utilizes a similar mechanism to imprint the effector-like gene signature. Based on the RNA sequencing (RNA-seq) analysis, *Tbx21* (the gene encoding T-bet) expression was modestly increased and *Eomes* expression was greatly diminished when IL-1β was administered (Fig. 2 A and Table S1). We measured similar changes in T-bet and Eomes protein levels that gave rise to higher T-bet/Eomes ratios (Fig. 2, D and E). Importantly, the T-bet/Eomes ratio correlated nicely with Gzm B expression during T cell priming in the draining LNs in the context of IL-1β treatment (Fig. 2, F and G), suggesting a key role of the T-bet/Eomes balance in controlling the IL-1β–driven effector-like gene signature.

The T-bet^hi^Eomes^lo^ phenotype has been associated with short-lived effector cells (Joshi et al., 2007). Despite having a similar phenotype, IL-1β–exposed OT-I cells showed increased expression of pro-survival genes such as *Il7r* and *Bcl2* (Fig. 2 A and Table S1), and exhibited long-term persistence and the ability to respond to antigen reencounter (Fig. S1, E–G). In sum, these findings suggest that the IL-1β–driven Gzm B induction is linked to an effector-like differentiation program controlled by T-bet and Eomes activities; such effector-like status does not resemble terminal differentiation but rather retains the memory potential.

### IL-1β induces local T cell accumulation in peripheral tissues

T cell responses generally initiate in the draining LNs, where T cell activation and expansion take place, followed by T cell egress from the LNs into circulation and subsequent tissue distribution. The discrepancy between the effects of IL-1β administration on T cell number increase in the draining LNs and peripheral tissues suggests a spatiotemporal regulation of T cell abundance by IL-1β.

**Figure 3.**
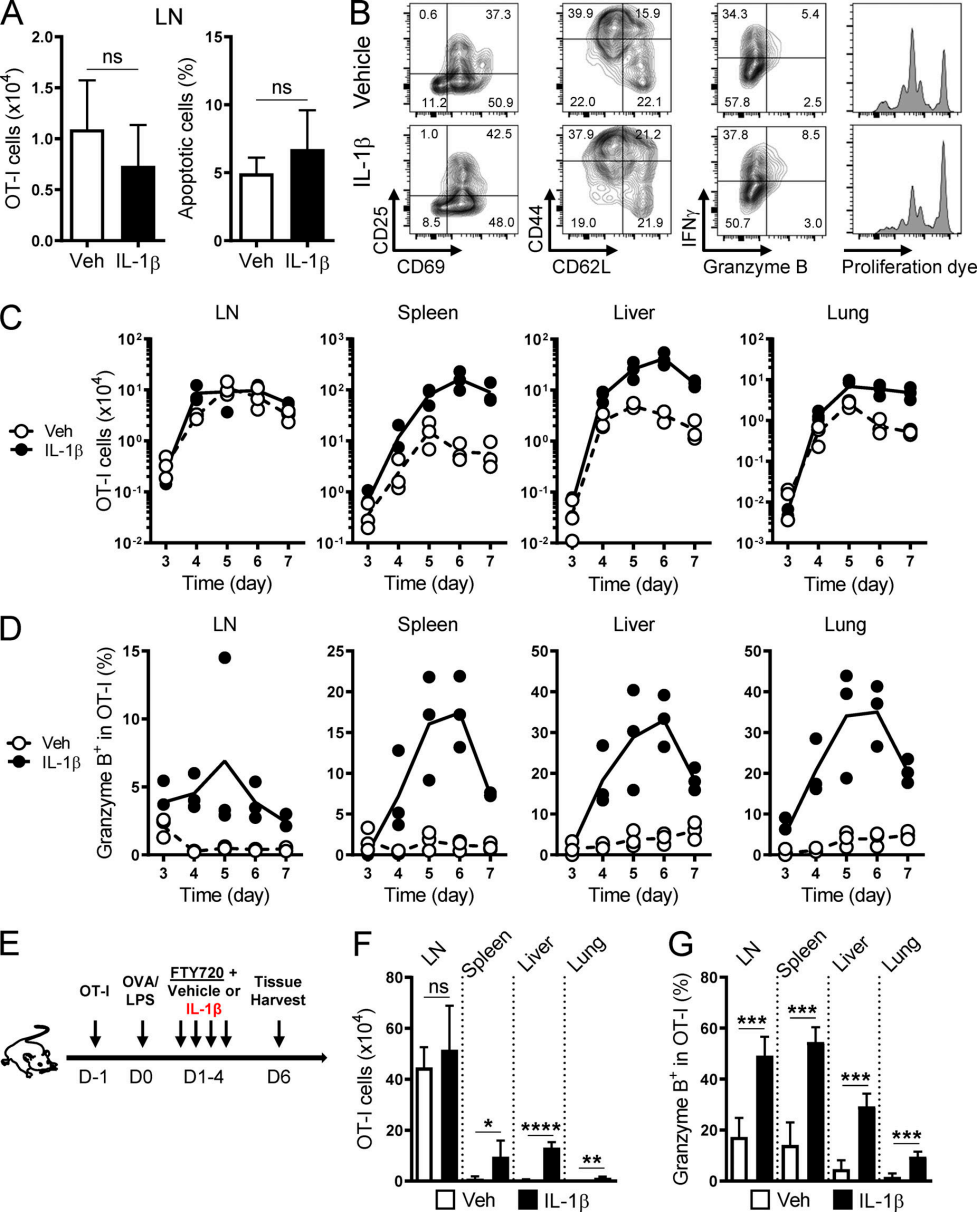
**Differential enhancements of T cell tissue accumulation and function by IL-1β. (A)** Absolute cell number and frequency of apoptotic cells (viability dye^+^ population) of vehicle (white bars)– and IL-1β (black bars)–exposed OT-I cells in the draining LNs on day 2 (*n* = 5). **(B)** Representative contour plots and histograms showing the expression of indicated molecules and proliferation dye in vehicle- and IL-1β–exposed OT-I cells as described in A. Plots were gated on the live, CD8^+^Vα2^+^CD45.1^+^CD45.2^–^ population. **(C and D)** Absolute cell number and frequency of OT-I cells expressing Gzm B isolated from indicated tissues on days 3–7 from vehicle (white circles)– and IL-1β (black circles)–treated mice (*n* = 3). **(E)** A schematic illustrating the induction of a primary OT-I response by OVA/LPS. Naive OT-I cells were transferred to a congenic host on day −1, followed by OVA/LPS immunization on day 0 and four daily injections of FTY720 along with vehicle or IL-1β on days 1–4. Tissues were harvested on day 6. **(F and G)** Absolute cell number and frequency of OT-I cells expressing Gzm B isolated on day 6 from indicated tissues of vehicle (white bars)– and IL-1β (black bars)–treated mice as described in E (*n* = 5). Data are representative of three (A and B) or two (C–G) independent experiments (*, P < 0.05; **, P < 0.01; ***, P < 0.001; ****, P < 0.0001; ns, not significant; error bars, SD).

To gain a more comprehensive understanding of how T cell responses were orchestrated by IL-1β administration, we analyzed the kinetics of primary OT-I cell responses at multiple tissues. We found that IL-1β showed little impact on early (day 2) T cell priming in the draining LNs with regard to cell numbers, apoptosis (Fig. 3 A), activation (CD69, CD25, CD44, and CD62L), function (Gzm B and IFN-γ), and proliferation (Fig. 3 B). As OT-I cells underwent clonal expansion and then exited the LNs, we began to detect a cell number increase caused by IL-1β in the peripheral tissues (Fig. 3 C). By contrast, the IL-1β effect on Gzm B was widely seen almost throughout the course of the study across different tissues (Fig. 3 D), suggesting that different mechanisms regulate cell number increase and Gzm B induction.

To determine if the increased tissue accumulation resulted from increased T cell export from the draining LNs, we treated mice with an immunosuppressant—FTY720 (Fingolimod)—to inhibit T cell egress from LNs (Rivera et al., 2008; Fig. 3 E). FTY720 treatment did not mitigate the IL-1β enhancement of peripheral OT-I cell numbers (Fig. 3 F) or Gzm B expression (Fig. 3 G), indicating that the increase in tissue T cell numbers did not originate from the draining LNs.

Alternatively, IL-1β could enhance tissue accumulation by promoting the differentiation of circulating T cells into tissue-resident memory T (Trm) cells. Trm cells reside in the extra-lymphoid tissues and do not return into circulation (Schenkel and Masopust, 2014). Enhanced differentiation into Trm cells would be accompanied by a reduction of circulating T cells, but IL-1β administration increased the frequency of OT-I cells in the blood (Fig. S2 A). Furthermore, IL-1β did not significantly affect the expression of the Trm markers—CD69 and CD103—in both OT-I and Pmel-1 models (Fig. S2, B and C), indicating its lack of impact on Trm differentiation. Taken together, these data suggest that the IL-1β enhancement of T cell numbers takes effect locally in peripheral tissues.

### IL-1β–induced T cell accumulation stems from enhanced tissue trafficking and survival

**Figure 4.**
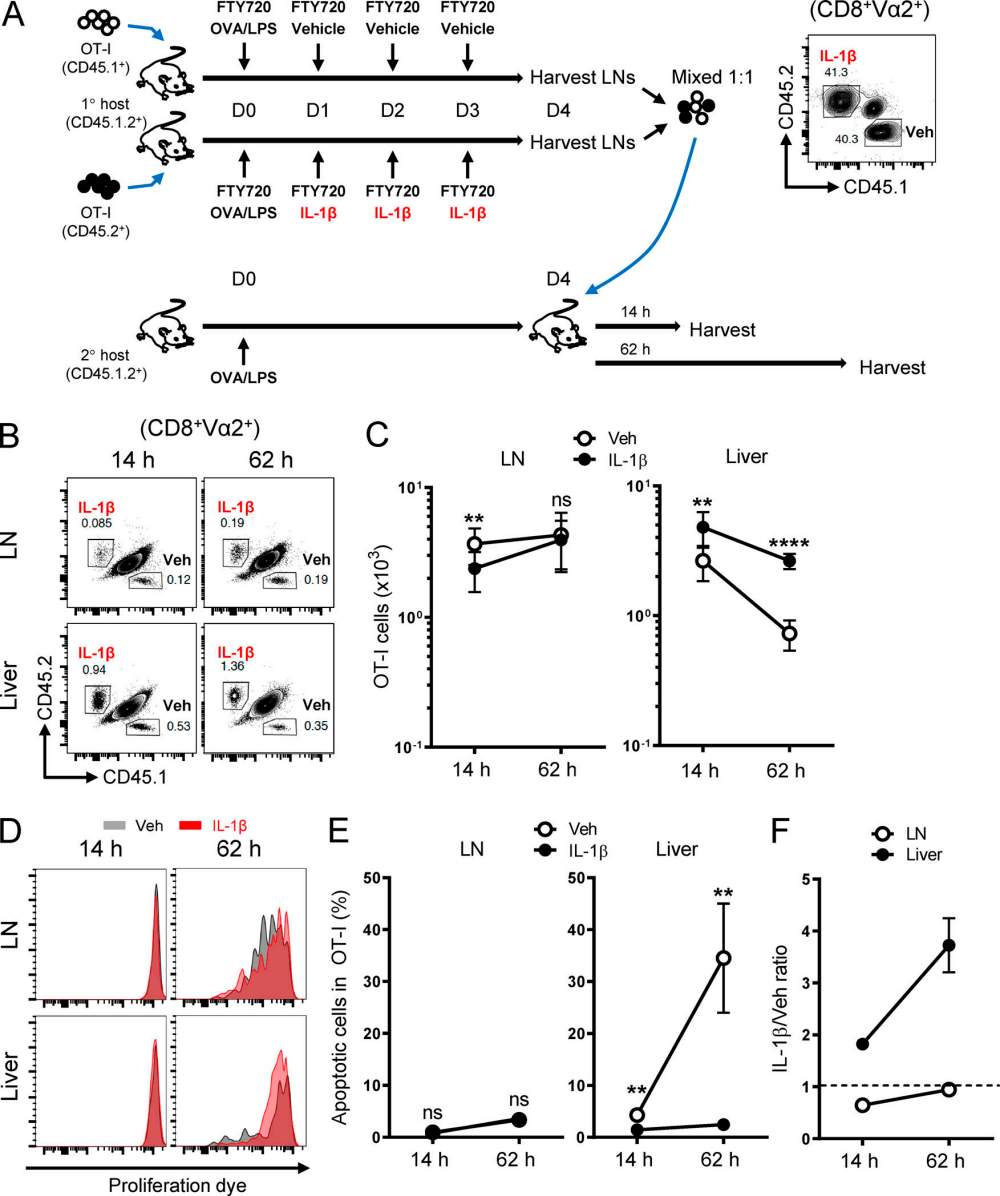
**IL-1β–exposed CD8^+^ T cells show superior trafficking and survival in peripheral tissues. (A)** A schematic illustrating the priming of adoptively transferred naive CD45.1^+^ OT-I cells and CD45.2^+^ OT-I cells by OVA/LPS under the influence of FTY720 in primary hosts (CD45.1^+^CD45.2^+^) treated with vehicle or IL-1β, respectively. Vehicle (CD45.1^+^)– and IL-1β (CD45.2^+^)–exposed OT-I cells were isolated on day 4 from the draining LNs, mixed at equal numbers (shown in the contour plot; CD8^+^Vα2^+^ gated), labeled with the eFluor 450 proliferation dye, and transferred to a secondary host that had been immunized with OVA/LPS 4 d earlier. Tissues were harvested 14 and 62 h after the second cell transfer (*n* = 5). **(B)** Representative contour plots showing the relative abundance of vehicle- and IL-1β–exposed OT-I cells in the draining LNs and liver 14 and 62 h after the transfer as described in A. Plots were gated on the live CD8^+^Vα2^+^ population. **(C)** Absolute cell number of the two OT-I populations in the draining LNs and liver as described in A. **(D)** Dilution of the eFluor 450 proliferation dye for vehicle (gray)– and IL-1β (red)–exposed OT-I cells in the draining LNs and liver as described in A. **(E)** Frequency of apoptotic cells (viability dye–positive and/or annexin V–positive populations) of the two OT-I populations in the draining LNs and liver as described in A. **(F)** Ratio of IL-1β– to vehicle-exposed OT-I cells in the draining LNs and liver 14 and 62 h after the transfer as described in A. Data are representative of two independent experiments (**, P < 0.01; ****, P < 0.0001; ns, not significant; error bars, SD).

T cell accumulation in peripheral tissues is a multifactorial process regulated by proliferation, survival, and trafficking. To determine the effects of IL-1β on these processes of T cells, we performed serial transfer experiments as illustrated in Fig. 4 A. Equal numbers of congenically marked vehicle- and IL-1β–exposed OT-I cells were isolated from the draining LNs of OVA-immunized primary hosts, labeled with cell proliferation dye, and cotransferred to a secondary OVA-immunized host that had not received IL-1β. We harvested the LNs and liver 14 and 62 h after cell transfer to evaluate the impact of IL-1β exposure in the primary host on the tissue trafficking, proliferation, and survival of OT-I cells in the secondary host.

Although started at a 1:1 ratio, IL-1β–exposed OT-I cells outnumbered the vehicle counterparts in the liver just 14 h after the cotransfer (Fig. 4, B and C). The cell number differences in the liver at the early time point were likely due to differential tissue trafficking because no alterations in cell divisions or apoptosis were observed between the populations (Fig. 4, D and E). The cell number differences in the liver became even more pronounced 62 h after cotransfer (Fig. 4, B, C, and F), likely not due to differential proliferation but rather caused by differential apoptosis (Fig. 4, D and E). By contrast, we only found a transient decrease in IL-1β–exposed OT-I cell abundance relative to vehicle-exposed counterparts in the LNs at 14 but not 62 h (Fig. 4, B and C) and measured no significant differences in proliferation or survival (Fig. 4, D and E). These data suggest that the IL-1β–mediated cell number increase is a cumulative outcome composed of enhanced T cell trafficking and survival in peripheral tissues.

### Administration of IL-1β directly and indirectly increases T cell numbers

**Figure 5.**
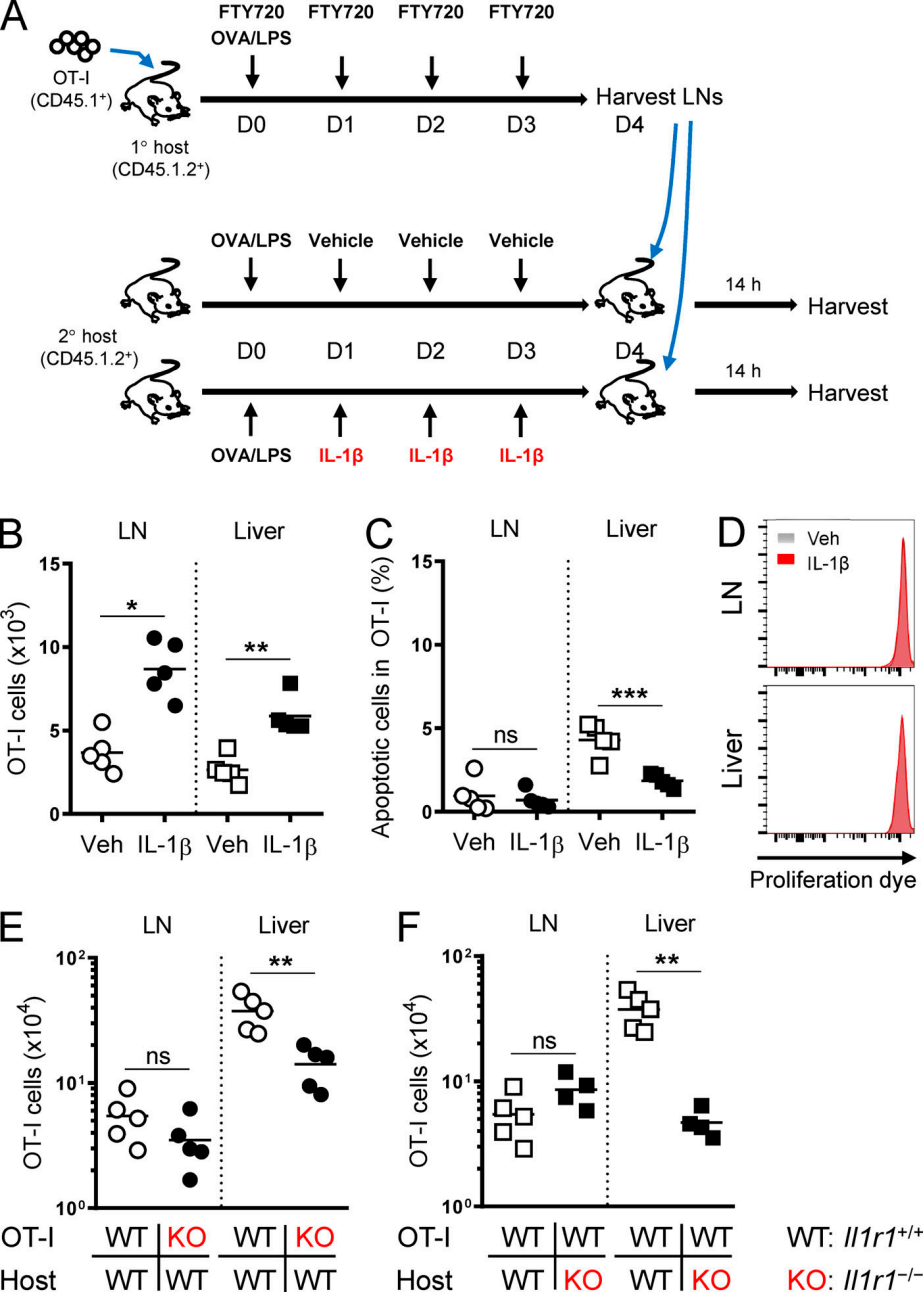
**Host-mediated enhancements of T cell tissue accumulation by IL-1β. (A)** A schematic illustrating the priming of adoptively transferred naive CD45.1^+^ OT-I cells by OVA/LPS under the influence of FTY720 in a primary host. OT-I cells were isolated on day 4 from the draining LNs, labeled with the eFluor 450 proliferation dye, and transferred to secondary hosts that had been treated with OVA/LPS and vehicle or IL-1β. Tissues were harvested 14 h after the second cell transfer (*n* = 5). **(B and C)** Absolute cell number and frequency of apoptotic OT-I cells in the draining LNs and liver from vehicle (open symbols)– and IL-1β (solid symbols)–treated hosts as described in A. **(D)** Dilution of the eFluor 450 proliferation dye for OT-I cells in the draining LNs and liver from vehicle (gray)– and IL-1β (red)–treated hosts as described in A. **(E)** Absolute cell number of WT (white circles) and *Il1r1*^−/−^ (KO; black circles) OT-I cells in the draining LNs and liver isolated on day 6 from WT hosts treated with OVA/LPS on day 0 and IL-1β on days 1–4 (*n* = 5). **(F)** Absolute cell number of WT OT-I cells in the draining LNs and liver isolated on day 6 from WT (white squares) and KO (black squares) hosts treated with OVA/LPS on day 0 and IL-1β on days 1–4 (*n* = 5). Data are representative of two (A–D) or four (E and F) independent experiments (*, P < 0.05; **, P < 0.01; ***, P < 0.001; ns, not significant).

IL-1 is known to induce tissue inflammation by acting on a wide range of cell types in the tissue microenvironment (Garlanda et al., 2013). Thus, we sought to determine the host contributions to the IL-1β effects on T cell numbers. To exclude the direct effects of IL-1β on T cells, we performed a serial transfer experiment as illustrated in Fig. 5 A. OT-I cells primed in the draining LNs without exogenous IL-1β were isolated from an OVA-immunized primary host and transferred to OVA-immunized secondary hosts that had been pretreated with either vehicle or IL-1β. In this setting, the last IL-1β injection was given to the secondary host 1 d before the OT-I transfer. Given the short half-life of recombinant IL-1β in vivo (Kudo et al., 1990), a direct exposure of OT-I cells to the residual exogenous IL-1β in the secondary host was unlikely. We found more OT-I cells in the liver and LNs from IL-1β–treated hosts compared with vehicle-treated hosts 14 h after the transfer (Fig. 5 B). These cell number differences were unlikely due to differential survival or proliferation (Fig. 5, C and D). Therefore, IL-1β may adapt the tissue microenvironment to facilitate T cell accumulation.

To more precisely determine the relative contributions of direct and indirect (host-mediated) effects of IL-1β on T cell numbers, we performed reciprocal adoptive transfer experiments using *Il1r1*^−/−^ (KO) OT-I cells and KO hosts. When transferred to a WT host treated with OVA/LPS and IL-1β, KO OT-I cells were unable to accumulate in the liver as efficiently as WT OT-I cells (Fig. 5 E). Furthermore, the accumulation of WT OT-I cells was severely impaired in the liver of KO hosts compared with WT hosts in the context of IL-1β treatment (Fig. 5 F). These findings indicate that IL-1R1 expression in both T cells and host cells is required for maximal enhancing effects of IL-1β on T cell numbers.

### Essential role of host cells in enhancing Gzm B expression in T cells by IL-1β administration

**Figure 6.**
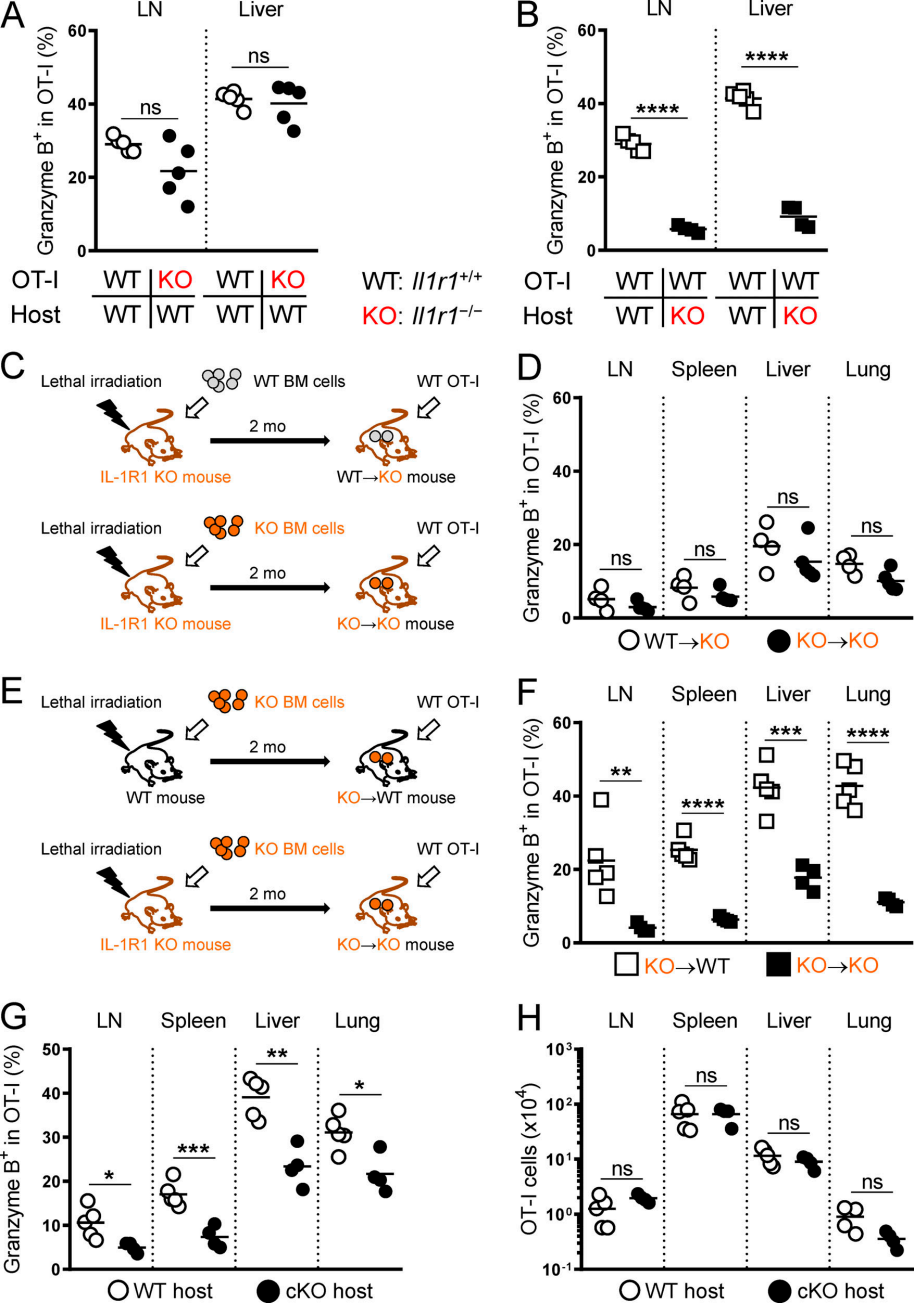
**IL-1β enhancement of T cell function is mediated by radio-resistant host cells. (A)** Frequency of WT (white circles) and *Il1r1*^−/−^ (KO; black circles) OT-I cells expressing Gzm B in the draining LNs and liver isolated on day 6 from WT hosts treated with OVA/LPS on day 0 and IL-1β on days 1–4 (*n* = 5). **(B)** Frequency of WT OT-I cells expressing Gzm B in the draining LNs and liver isolated on day 6 from WT (white squares) and KO (black squares) hosts treated with OVA/LPS on day 0 and IL-1β on days 1–4 (*n* = 5). **(C)** A schematic illustrating the generation of bone marrow (BM) chimeric mice. WT→KO and KO→KO mice were generated by reconstituting lethally irradiated *Il1r1*^−/−^ (KO) mice with WT or KO bone marrow cells, respectively. 2 mo later, WT OT-I cells were transferred to the bone marrow chimeric hosts, followed by OVA/LPS and IL-1β treatments. **(D)** Frequency of OT-I cells expressing Gzm B isolated on day 6 from WT→KO (white circles) and KO→KO (black circles) hosts treated with OVA/LPS on day 0 and IL-1β on days 1–4 (*n* = 4 or 5). **(E)** A schematic illustrating the generation of bone marrow chimeric mice. KO→WT and KO→KO mice were generated by reconstituting lethally irradiated WT or KO mice with KO bone marrow cells, respectively. 2 mo later, WT OT-I cells were transferred to the bone marrow chimeric hosts, followed by OVA/LPS and IL-1β treatments. **(F)** Frequency of OT-I cells expressing Gzm B isolated on day 6 from KO→WT (white squares) and KO→KO (black squares) hosts treated with OVA/LPS on day 0 and IL-1β on days 1–4 (*n* = 5 or 4). **(G and H)** Frequency of OT-I cells expressing Gzm B and absolute OT-I cell number isolated on day 6 from *Il1r1^fl/fl^Cdh5*-*Cre*^−^ (WT; white circles) and *Il1r1^fl/fl^Cdh5*-*Cre*^+^ (cKO; black circles) hosts treated with OVA/LPS on day 0 and IL-1β on days 1–4 (*n* = 5). Data are representative of four (A and B), two (C–F), or three (G and H) independent experiments (*, P < 0.05; **, P < 0.01; ***, P < 0.001; ****, P < 0.0001; ns, not significant).

In contrast to the cell number effects of IL-1β, the Gzm B induction did not require T cell expression of IL-1R1 as KO OT-I cells showed a comparable frequency of Gzm B^+^ cells to WT OT-I cells (Fig. 6 A). However, Gzm B induction was abrogated when host cells were unable to be stimulated by IL-1β (Fig. 6 B), indicating that the Gzm B effect of IL-1β was mediated through host cells. To further determine if radio-sensitive host cells were responsible for this effect, we generated bone marrow chimeric mice by reconstituting lethally irradiated KO mice with either WT or KO bone marrow cells. 2 mo later, we analyzed the impact of IL-1β on transferred WT OT-I cells in these bone marrow chimeric hosts (Fig. 6 C). IL-1R1 expression in radio-sensitive host cells was insufficient to trigger Gzm B expression (Fig. 6 D). To examine the contribution of radio-resistant host cells, we reconstituted lethally irradiated WT and KO mice with KO bone marrow cells (Fig. 6 E). IL-1R1 expression in radio-resistant host cells restored the Gzm B induction (Fig. 6 F). Based on these bone marrow chimera experiments, we concluded that IL-1β acts on radio-resistant host cells to promote Gzm B expression in T cells.

On the other hand, IL-1R1 expression in neither radio-sensitive nor radio-resistant host compartment alone was able to substantially increase the numbers of transferred OT-I cells in the peripheral tissues (Fig. S3, A and B). It is conceivable that multiple host cell types of both hematopoietic and nonhematopoietic origins are involved in the cell number–enhancing effects of IL-1β.

A previous report showing the ability of liver sinusoidal endothelial cells to induce Gzm B expression in CD8^+^ T cells (Böttcher et al., 2014) prompted us to investigate the role of endothelial cells in IL-1β–driven Gzm B induction. We conditionally disrupted the *Il1r1* gene in vascular endothelial cells by introducing the *Cdh5* (cadherin 5, also known as vascular endothelial cadherin)–*Cre* transgene (Chen et al., 2009) to *Il1r1^fl/fl^* mice. Gzm B expression in transferred OT-I cells was substantially decreased in these IL-1R1 conditional KO (cKO) hosts compared with WT littermate controls (Fig. 6 G), whereas OT-I cell numbers were unaffected (Fig. 6 H). These results indicate that cells that had expressed *Cdh5* were critical for the IL-1β–driven Gzm B induction but not the cell number increase.

TCR activation is a strong stimulus for Gzm B expression (Kelso et al., 2002). To investigate if IL-1β–stimulated radio-resistant host cells induced Gzm B expression in T cells through TCR activation, we transferred WT bone marrow cells to lethally irradiated *B2m*^−/−^ mice (Fig. S3 C). Radio-resistant cells in these bone marrow chimeric mice lacked the ability to activate OT-I TCR, but the IL-1β–driven Gzm B expression in transferred OT-I cells was unaffected (Fig. S3 D). We also did not observe any major, tissue-wide changes in cell numbers (Fig. S3 E). Altogether, these data suggest that IL-1β–stimulated radio-resistant host cells enhance Gzm B expression in T cells in a TCR-independent fashion.

### IL-1β–induced IL-6 and IL-12 are dispensable for Gzm B induction

**Figure 7.**
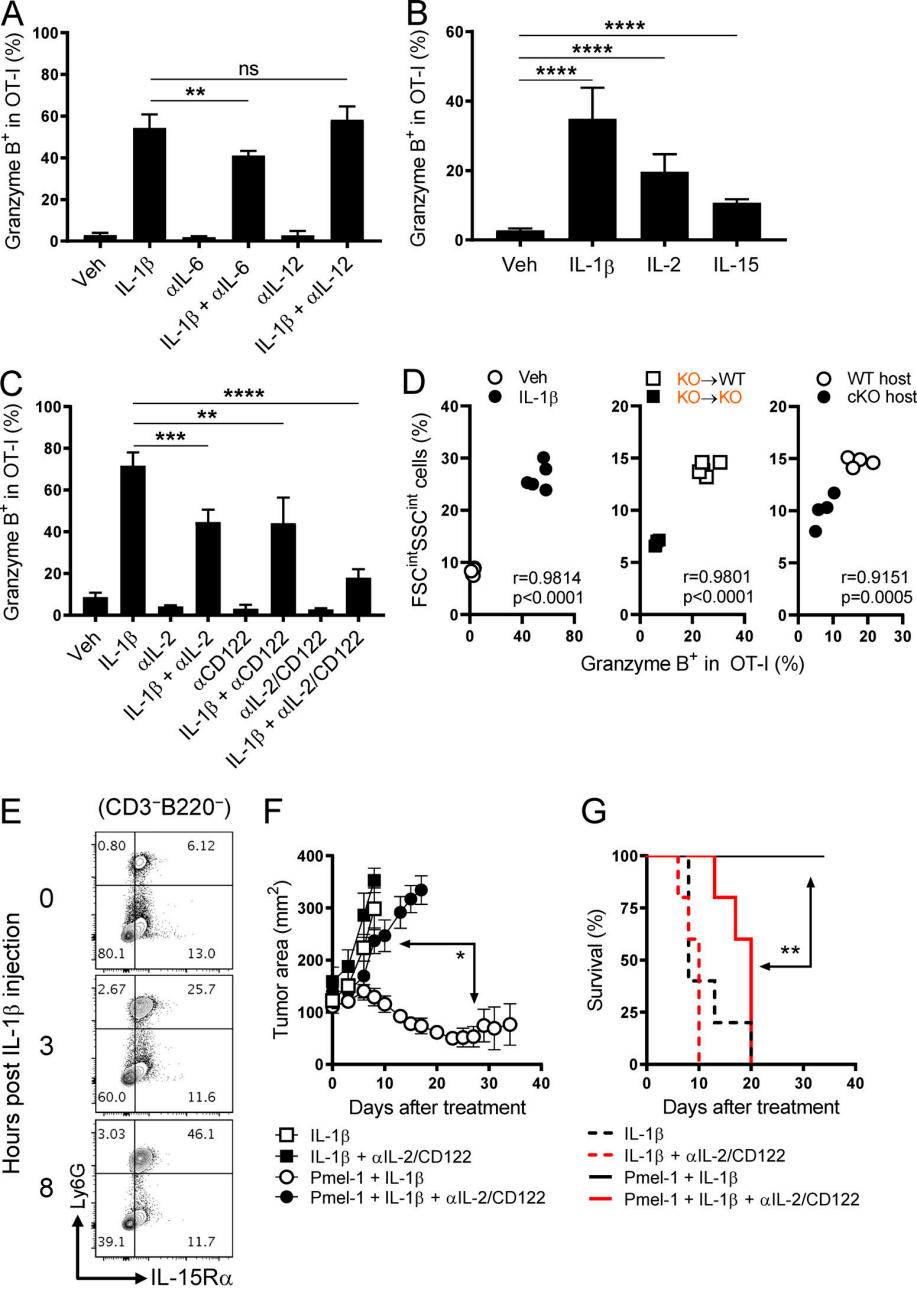
**IL-1β enhancement of T cell function depends on IL-2 and IL-15 but not IL-6 or IL-12. (A–C)** Frequency of OT-I cells expressing Gzm B isolated on day 6 from the spleen of mice injected with OVA/LPS on day 0 and indicated treatments on days 1–4 (*n* = 5). **(D)** Correlation between the frequency of OT-I cells expressing Gzm B and the frequency of FSC^int^SSC^int^ population in the experiments shown in A (left), Fig. 6 F (middle), and Fig. 6 G (right). Pearson’s correlation coefficient (r) and P value are shown. **(E)** Representative contour plots showing Ly6G and IL-15Rα expression in the spleen at 0, 3, and 8 h after a single injection of IL-1β (2 µg). Plots were gated on the live, CD3^−^B220^–^ population. **(F and G)** Tumor area and overall survival of tumor-bearing mice that received PBS or Pmel-1 cells on day 0 and other indicated treatments on days 0–4 (*n* = 5). Data are representative of two (A, B, and E–G) or three (C) independent experiments (*, P < 0.05; **, P < 0.01; ***, P < 0.001; ****, P < 0.0001; ns, not significant; error bars, SD for A–C; SEM for F).

Having shown that the IL-1β–driven Gzm B induction did not require the engagement of IL-1R or TCR on adoptively transferred CD8^+^ T cells, we next sought to determine if the inflammatory cytokines induced by IL-1β triggered Gzm B expression. Upon a single injection of IL-1β, we were able to detect IL-6, IL-12, and TNFα in the serum with different kinetics (Fig. S4 A). Despite the reported positive effects of IL-6 and IL-12 on Gzm B expression in CD8^+^ T cells (Chowdhury et al., 2011; MacLeod et al., 2011; Böttcher et al., 2014; Rubinstein et al., 2015), we only saw a modest reduction in Gzm B expression with anti–IL-6 neutralizing antibody and no effect with anti–IL-12 neutralizing antibody when compared with IL-1β alone (Fig. 7 A). Moreover, blocking these cytokines had no or negligible effect on the IL-1β–induced cell number increase (Fig. S4 B). Therefore, despite being induced promptly by IL-1β, IL-6 and IL-12 are unlikely to mediate its Gzm B–enhancing effects on T cells.

### IL-1β enhancement of T cell responses depends on IL-2 and IL-15

In vitro studies have shown that IL-2 and IL-15 can induce Gzm B expression in CD8^+^ T cells without TCR stimulation (Tamang et al., 2006). Because GSEA of the RNA-seq data (Fig. 2 A) indicated that IL-2 and IL-15 pathways were more activated in IL-1β–exposed OT-I cells compared with vehicle-exposed counterparts (Fig. S4, C and D), we hypothesized that IL-2 and IL-15 were involved in the IL-1β–driven Gzm B induction in CD8^+^ T cells.

We found that administration of recombinant IL-2 or IL-15 augmented Gzm B expression in vivo (Fig. 7 B). To investigate if the Gzm B effect of IL-1β would be negated by blocking IL-2 and IL-15, we used antibodies targeting IL-2 and IL-2/15Rβ (CD122), respectively. Although CD122 is shared by IL-2R and IL-15R complexes, anti-CD122 antibody (clone TM-β1) has little effect on IL-2 in vitro (François et al., 1996) and potent blocking efficiency on IL-15 in vivo (François et al., 1996; Yokoyama et al., 2009). We found that blocking IL-2 or IL-15 partially reduced the Gzm B augmentation by IL-1β; however, blocking both cytokines nearly abrogated the induction of Gzm B (Fig. 7 C). This suggests a critical role of both IL-2 and IL-15 in enhancing T cell functionality by IL-1β.

In addition to T cell functionality, we also investigated the role of IL-2 and IL-15 in the IL-1β–induced T cell number increase. Administration of recombinant IL-2 or IL-15 resulted in increased OT-I cell numbers (Fig. S4 E), but only IL-15 blockade partially mitigated the IL-1β–induced cell number increase (Fig. S4 F). Furthermore, in both adoptively transferred OT-I and Pmel-1 cells, we found that IL-1β administration led to an elevated expression of IL-7Rα (CD127), a marker highly associated with T cell survival (Kaech et al., 2003; Martin et al., 2013; Fig. S4, G and H). These findings were consistent with RNA-seq data (Fig. 2 A) and the enhanced survivability in peripheral tissues (Fig. 4 E). Together, these results reveal that the effect of IL-1β on Gzm B induction depends on IL-2 and IL-15, and the effect on cell number increase depends on IL-15, IL-1β itself (Fig. 5 E), and likely IL-7.

Despite the important role of IL-2 and IL-15 in the IL-1β–driven Gzm B induction, IL-2 was not readily induced by IL-1β administration at both the mRNA (Fig. S5 A) and protein levels (Fig. S5 B). Moreover, IL-1β neither made CD8^+^ T cells more sensitive to IL-2 by increasing the expression of IL-2Rα (CD25) or CD122 (Fig. S5, C and D) nor reduced the competition for IL-2 by lowering the frequency or CD25 expression of T reg cells (Fig. S5, E and F). IL-15 signaling is primarily triggered by the binding of IL-15/IL-15Rα complex on the surface of an opposing cell to IL-15Rβ/γc complex on the surface of a T cell (Stonier and Schluns, 2010). In contrast to IL-2, we found that IL-1β increased the mRNA levels of *Il15* and *Il15ra* in the spleen (Fig. S5 G). Therefore, despite the dependence on both IL-2 and IL-15, IL-1β likely augments Gzm B expression by increasing the availability of IL-15, but not IL-2, to CD8^+^ T cells.

In search of cellular sources of the additional IL-15 signaling, we observed a pronounced expansion of a cell population in the spleen of IL-1β–treated mice with increased cell size and granularity (FSC^int^SSC^int^) relative to lymphocytes, particularly CD11b^+^Gr-1^+^ myeloid cells (Fig. S5 H). Retrospectively, we found a strong correlation between the frequency of this cell population and the frequency of OT-I cells expressing Gzm B (Fig. 7 D) in the following experiments: (1) vehicle- and IL-1β–treated WT hosts as described in Fig. 7 A, (2) IL-1β–treated KO→WT and KO→KO bone marrow chimeric hosts as described in Fig. 6 F, and (3) IL-1β–treated WT and cKO mice as described in Fig. 6 G. To further characterize the CD11b^+^Gr-1^+^ population induced by IL-1β administration, we found a rapid and selective expansion of neutrophils (CD11b^+^Ly6G^+^Ly6C^int^) in the spleen (Fig. S5 I) with high surface IL-15Rα expression (Fig. 7 E and Fig. S5 J). These findings implicate a possible role of neutrophils in the IL-1β–induced Gzm B elevation through the trans-presentation of IL-15 to T cells.

Having shown the impact of IL-2 and IL-15 dual blockade on IL-1β enhancement of T cell numbers and Gzm B expression, we next evaluated its effect on the antitumor function. Blocking IL-2 and IL-15 activities in the context of IL-1β administration completely abrogated the ability of Pmel-1 cells to control tumor growth and shortened the life span of tumor-bearing mice (Fig. 7, F and G), suggesting a strong dependence of IL-1β enhancement of T cell antitumor function on IL-2 and IL-15. Collectively, these findings reveal a novel link between IL-1β and IL-2/IL-15 in modulating the antitumor function of CD8^+^ T cells.

## Discussion

We demonstrate here that the administration of IL-1β can potentiate the efficacy of adoptively transferred antitumor T cells to mediate tumor regression. We found that IL-1β increased both the cell numbers and functionality of antigen-specific T cells, with each effect being mediated through separate in vivo mechanisms. IL-1β engaged both T cells and host cells to enhance T cell tissue trafficking and survival, leading to elevated tissue accumulation. By contrast, IL-1β–mediated augmentation of T cell functionality was driven through IL-1R on radio-resistant host cells and depended on IL-2 and IL-15 but not IL-6, IL-12, or TCR stimulation.

We used the *Cdh5-Cre* model to determine if the radio-resistant host cells mediating the Gzm B effect of IL-1β were of the endothelial lineages. While the *Cdh5-Cre* induces the *Il1r1* deletion in endothelial cells, this gene deletion is inherited by hematopoietic cells as a consequence of these cells having originated from the endothelial cell lineages (Zovein et al., 2008; Chen et al., 2009). Other commonly used endothelial-specific Cre models such as *Tie2-Cre* and *Flk1-Cre* have also been reported to show Cre activity in hematopoietic cells (Payne et al., 2018). Consequently, although the data from *Il1r1^fl/fl^Cdh5-Cre* mice support the involvement of endothelial cells in IL-1β enhancement of T cell functionality, we cannot rule out a potential role of radio-resistant hematopoietic cells such as tissue-resident macrophages (Gordon and Plüddemann, 2017). An in vivo model that would allow for the isolation of the IL-1β effect on vascular endothelial cells from their derivatives needs to be established in order to unambiguously determine the immediate target of IL-1β.

The strong correlation between the abundance of FSC^int^SSC^int^ cells and the frequency of OT-I cells expressing Gzm B implicate these cells in enhancing T cell functionality. Initial analyses identified CD11b^+^Gr-1^+^ cells as the main contributor of these cells. Based on Ly6C and Ly6G expression, these myeloid cells can be further divided into Ly6C^hi^Ly6G^–^ inflammatory monocytes, Ly6C^lo^Ly6G^–^ tissue-resident monocytes, and Ly6C^int^Ly6G^+^ neutrophils (Hey et al., 2016). We found that the Ly6C^int^Ly6G^+^ neutrophils were selectively recruited to the spleen following the administration of IL-1β and may serve as a source of IL-15 signaling for CD8^+^ T cells because of the high surface IL-15Rα expression. Similar to macrophages, neutrophils can be polarized by environmental cues to acquire an antitumor (N1) or pro-tumor (N2) phenotype (Powell and Huttenlocher, 2016; Nicolás-Ávila et al., 2017). The ability of neutrophils to enhance the antitumor function of CD8^+^ T cells in the context of IL-1β administration warrants further investigation.

Despite its great potency in improving the activity of adoptively transferred antitumor T cells, IL-1β can cause acute toxicities at high doses. We found that IL-1β caused severe morbidity and mortality in mice when administered subcutaneously at a daily dose of 80 µg/kg for 5 d consecutively. Recent studies in mice have also suggested a crucial role of host-derived IL-1 in the pathogenesis of cytokine release syndrome induced by chimeric antigen receptor–T cells (Giavridis et al., 2018; Norelli et al., 2018). These findings raise important safety concerns regarding the use of exogenous IL-1β in the setting of ACT. In our animal models, we were able to find reduced doses (60 µg/kg/d for 4 d or 40 µg/kg/d for 5 d) that caused only limited toxicity and no deaths in mice. IL-1β in combination with Pmel-1 cells not only induced the regression of established B16 melanomas but also prolonged the survival of tumor-bearing mice. This indicates that no dose-limiting toxicity was observed in these animals at doses that were effective. In humans, early clinical trials showed that the toxicities of IL-1 were dose-related and manageable (Veltri and Smith, 1996). However, careful and slow dose escalation studies are required to assess the safety of using IL-1 in conjunction with ACT before addressing its antitumor potency. Furthermore, agents that block the production or activity of IL-6, TNFα, and nitric oxide may also be studied to further mitigate any toxicities of IL-1.

The role of IL-1 in tumorigenesis is still debatable as it can induce the expression of pro-angiogenesis and pro-metastatic factors (Lewis et al., 2006; Dinarello, 2010; Voronov et al., 2014), while antitumor activity has also been reported for IL-1 (Zöller et al., 1992; Grandis et al., 1995; Kim et al., 2015; Le et al., 2016). In our subcutaneous B16 melanoma model, IL-1β injections alone did not significantly alter the growth of established tumors or mouse mortality. When used in combination with ACT, IL-1β enhanced the accumulation and effector function of adoptively transferred tumor-reactive CD8^+^ T cells at tumors, resulting in improved tumor regression and mouse survival. While naive or less differentiated T cells are more desirable for ACT (Gattinoni et al., 2005b), they heavily rely on proper activating signals in vivo for the differentiation into cytolytic effector cells and infiltration into tumors. We propose that IL-1β administration conditions the host environment to provide these signals to adoptively transferred antitumor T cells, thereby improving their efficacy to eradicate tumors. Although IL-1 as a monotherapy showed little antitumor activity against several malignancies in early clinical trials (Veltri and Smith, 1996), it can potentially be used in combination with ACT to increase the response rates for cancers that do not respond to current ACT treatments.

## Materials and methods

### Mice and cell line

Mice of the following strains were obtained from Taconic Farms: C57BL/6 OT-I *Rag2*^−/−^ CD45.1, C57BL/6 OT-I *Rag2*^−/−^ CD45.2, C57BL/6 OT-I *Rag2*^−/−^ *Il1r1*^−/−^ CD45.2, C57BL/6, C57BL/6 CD45.1/2, and C57BL/6 *Il1r1*^−/−^. *Cdh5-Cre*, *B2m^tm1Unc^*, and C57BL/6 Pmel-1 Thy1.1 mice were purchased from The Jackson Laboratory. *Il1r1^fl/fl^* mice on a C57BL/6 background were obtained from Werner Muller (University of Manchester, Manchester, UK; Abdulaal et al., 2016) and crossed with *Cdh5-Cre* mice for at least five generations before being used for experiments. *Il1r1^fl/fl^Cdh5-Cre*^+^ (cKO) and *Il1r1^fl/fl^Cdh5-Cre*^−^ (WT) littermate controls were used in the experiments. All mice were maintained under specific pathogen-free conditions under protocols approved by the National Institute of Allergy and Infectious Diseases and National Cancer Institute Animal Care and Use Committees and were used for experiments at 6–16 wk of age.

The B16-mhgp100 melanoma cell line was retrovirally transduced to overexpress the modified mouse gp100 with glutamate-glycine-serine residues replaced by human residues lysine-valine-proline at positions 25–27 (Hanada et al., 2019) and was maintained in DMEM with 10% FBS, 1% glutamine, 1% penicillin-streptomycin, and 10 µg/ml of blasticidin (InvivoGen).

### Cytokines, antibodies, and flow cytometry

Recombinant mouse IL-1β was obtained from Paul Wingfield (National Institues of Health, Bethesda, MD; Wingfield et al., 1986). Recombinant human IL-2 was obtained from Chiron Corp. Recombinant human IL-15 was obtained from PeproTech. Fluorochrome-conjugated anti-CD11b (M1/70), anti-Ly6G (1A8), anti-NK1.1 (PK136), anti–T-bet (O4-46), and FITC– and APC–annexin V were obtained from BD Biosciences. Fluorochrome-conjugated anti-Thy1.2 (53–2.1) and anti-Eomes (Dan11mag) were obtained from Affymetrix. Fluorochrome-conjugated anti-B220 (RA3-6B2), anti-CD3ε (145-2C11), anti-CD4 (GK1.5), anti-CD8 (53–6.7), anti-CD11c (N418), anti-CD25 (PC61), anti-CD44 (IM7), anti-CD45.1 (A20), anti-CD45.2 (104), anti-CD62L (MEL-14), anti-CD69 (H1.2F3), anti-CD103 (2E7), anti-CD122 (5H4), anti-CD127 (A7R34), anti–Gr-1 (RB6-8C5), anti–IFN-γ (XMG1.2), anti-Ly6C (HK1.4), anti-Thy1.1 (OX-7), anti-TNFα (MP6-XT22), anti-Vα2 (B20.1), anti-Vβ5 (MR9-4), and anti-Vβ13 (MR12-3) were obtained from BioLegend. Fluorochrome-conjugated anti-Gzm B (GB11) and anti-Foxp3 (FJK-16s) were obtained from Thermo Fisher Scientific. Fluorochrome-conjugated anti–IL-15Rα (888220) was obtained from R&D Systems. Anti–FcγR II (2.4G2) was obtained from Harlan (Indianapolis, IN). Anti-mouse IL-2 (JES6-5H4), anti-mouse IL-6 (MP5-20F3), anti-mouse IL-12 (R2-9A5), and anti-mouse CD122 (TM-β1) monoclonal antibodies were obtained from BioXCell.

For surface staining, cells were incubated with 100 µl of PBS containing anti-FcγR II (2.4G2), fixable viability dye eFluor 780 (1:1,000; Affymetrix), and fluorochrome-conjugated antibodies at 4°C for 30 min. For cytokine intracellular staining, cells were first stimulated with PMA (50 ng/ml) and ionomycin (500 ng/ml) in the presence of GolgiStop (1:1,500; BD Biosciences) for 4–6 h at 37°C, followed by surface staining, fixation/permeabilization using BD Cytofix/Cytoperm Kit (BD Biosciences), and intracellular staining with fluorochrome-conjugated antibodies at 4°C overnight. For Gzm B and transcription factor intracellular staining, cells were surface-stained, fixed/permeabilized using Foxp3 Transcription Factor Staining Buffer Set (Affymetrix), and intracellular-stained with fluorochrome-conjugated antibodies at 4°C overnight. All samples were analyzed using a BD LSRFortessa or LSR II flow cytometer (BD Biosciences) and FlowJo software (Tree Star).

Proliferation of OT-I cells was measured by incubating cells with 10 µM of the Cell Proliferation Dye eFluor 450 (Thermo Fisher Scientific) in PBS at 10 × 10^6^ cells/ml at 37°C for 10 min, followed by a 5-min incubation on ice. The cells were washed three times with RPMI 1640 containing 5% FBS. The labeling of OT-I cells with the Cell Proliferation Dye eFluor 450 was verified by flow cytometry before adoptive transfer.

### Adoptive transfer, in vivo priming of OT-I cells, and administration of cytokines and monoclonal antibodies

Unless otherwise indicated, naive (CD44^lo^CD62L^hi^) OT-I cells were enriched from the spleen and LNs of donor mice using a naive CD8α^+^ T cell isolation kit (Miltenyi) and injected i.v. to recipient mice (10^4^ cells/mouse) on day −1. Mice were immunized s.c. with endotoxin-free OVA protein (100 µg; BioVendor) and LPS (25 µg; InvivoGen) on day 0. Vehicle (1× PBS), IL-1β (1.5 µg/injection), IL-2 (2 × 10^5^ IU or 12 µg/injection), or IL-15 (1.5 µg/injection) was injected s.c. daily on days 1–4. In some experiments, anti–IL-2, anti–IL-6, anti–IL-12, and/or anti-CD122 monoclonal antibodies (250 µg/injection) were injected i.p. daily on days 1–4.

### Enrichment of lymphocytes from liver and lung

Isolation of lymphocyte populations from the liver and lung was previously described (Ben-Sasson et al., 2013a). In brief, the liver was mechanically homogenized to make single-cell suspensions, followed by a centrifugation at 30 ×*g* for 3 min to remove hepatocytes at the bottom. The remaining cells were washed, resuspended in 33% isotonic Percoll solution, and spun at 500 ×*g* for 15 min at room temperature. The pellet was treated with ACK lysis buffer to remove RBCs, washed, and resuspended in RPMI 1640 containing 10% FCS.

The lung was mechanically homogenized and digested with collagenase/DNase I at 37°C for 30 min to make single-cell suspension. The lung digest was washed, resuspended in 40% isotonic Percoll solution, and spun at 2,000 RPM for 20 min at room temperature. The pellet was treated with ACK lysis buffer to remove RBCs, washed, and resuspended in RPMI 1640 containing 10% FCS.

### Adoptive immunotherapy for cancer

C57BL/6 mice were implanted s.c. with B16-mhgp100 cells (5 × 10^5^ cells) 10–12 d before treatment. Tumor masses were allowed to become well established and highly vascularized until they achieved a diameter of ∼1 cm. 1 d before the Pmel-1 cell transfer, tumor-bearing mice were sub-lethally irradiated (600 cGy). The next day, the mice were randomized and injected i.v. with 10^5^ naive Pmel-1 CD45.1 cells. Five daily s.c. injections of vehicle or IL-1β (1.0 µg/injection) were given starting from the day of cell transfer. Tumor area was measured by an independent investigator in a blinded manner every 2 or 3 d after treatment and was determined as a product of its length and width. Mice with tumors approaching 400 mm^2^ were euthanized for humane reasons.

### Generation of bone marrow chimeras

Recipient mice were lethally irradiated at 550 cGy twice with a 3-h interval before receiving 4–5 × 10^6^ bone marrow cells from donor mice. Trimethoprim and sulfamethoxazole were added to drinking water and changed every week for 5 wk after bone marrow transplantation. Hematopoietic reconstitution was confirmed by staining PBLs from bone marrow chimeras with anti-CD45.1 and anti-CD45.2 antibodies 2 mo after bone marrow transplantation.

### Measurements of serum IL-6, TNFα, and IL-12 p70 levels

Submandibular blood samples collected from mice were placed in BD Microtainer Serum Separator Tubes (BD Biosciences) and stored at room temperature for at least 30 min. The tubes were then spun at 5,000 RPM for 5 min, and the serum was collected and used in a Mouse IL-6 ELISA Ready-Set-Go! Kit, Mouse TNFα ELISA Ready-Set-Go! Kit (Thermo Fisher Scientific) and Mouse IL-12 p70 Quantikine ELISA Kit (R&D Systems) following the manufacturers’ instructions.

### RNA-seq analysis and real-time PCR

For RNA-seq, total RNA was extracted from OT-I cells isolated on day 4 from the draining LNs of mice treated with vehicle or IL-1β using the RNeasy Plus Mini Kit (Qiagen). RNA integrity was measured using High Sensitivity RNA ScreenTape assessed using TapeStation 2200 (Agilent). The RNA Integrity Number was greater than eight for all the samples. For genome-scale transcriptome analysis, 200 ng of total RNA was used to prepare the RNA-seq library using the TruSeq RNA sample preparation kit (Illumina). Paired-end RNA-seq was performed on a HiSeq 2500 platform at the National Cancer Institute’s Center for Cancer Research Sequencing Facility (Illumina). Sequenced reads were aligned to the mouse genome (mm10) using Spliced Transcripts Alignment to a Reference (version 2.4.2a). Subread (version 1.4.6) was used to count the number of reads mapped to each gene. Genes with reads less than five across all samples were removed, and the data were normalized using the Trimmed Mean of M-values normalization. Differential gene expression values were computed using EdgeR (Bioconductor). To define differentially expressed genes, we used a twofold change and P < 0.05 difference between groups. GSEA was performed using GSEA v2.2.3 from The Broad Institute using a gene list ranked on fold change in expression between IL-1β and vehicle control groups. GSEA was run using molecular signature database v.5.2 C7 Immunological signature gene sets (Subramanian et al., 2005). All raw data files have been deposited in the Gene Expression Omnibus repository under accession no. GSE127234.

For real-time PCR, total RNA was extracted from splenocytes isolated from vehicle- and IL-1β–treated mice using the RNeasy Plus mini kit (Qiagen). cDNA was generated using high-capacity cDNA reverse transcription kit (Thermo Fisher Scientific). Real-time RT-PCR was performed for all genes with primers from Applied Biosystems by Prism 7900HT (Applied Biosystems). Gene expression was calculated relative to *Actb* expression.

### Statistics

A two-tailed Student’s *t* test was used to compare the two groups for all experiments (except for Fig. 1, E and F; and Fig. 7, D, F, and G) using GraphPad Prism 8 software (GraphPad Software). For Fig. 1 E and Fig. 7 F, tumor areas were plotted as the mean ± SEM for each data point, and tumor growth curves were compared using the Wilcoxon rank sum test. For Fig. 1 F and Fig. 7 G, the Mantel–Cox log-rank test was used to compare the survival curves using GraphPad Prism 8 software. For Fig. 7 D, the Pearson correlation test was used to calculate the correlation between the frequency of the FSC^int^SSC^int^ population and the frequency of OT-I cells expressing Gzm B using GraphPad Prism 8 software.

### Online supplemental material

Fig. S1 shows the schematics of OT-I and Pmel-1 models, the effects of IL-1β administration on endogenous SIINFEKL-tetramer^+^ CD8^+^ T cells, and the impact of IL-1β administration on a recall response. Fig. S2 shows the frequency of OT-I cells in the blood and CD69/CD103 expression in OT-I and Pmel-1 cells. Fig. S3 shows OT-I cell numbers in the bone marrow chimera experiments described in Fig. 6 and OT-I data for the bone marrow chimera experiment using β_2_M KO mice. Fig. S4 shows serum levels of IL-6, IL-12, and TNFα upon IL-1β administration, OT-I cell numbers in the experiments described in Fig. 7, GSEA of IL-1β–exposed versus vehicle-exposed OT-I cells, and the impact of IL-1β administration on CD127 expression in OT-I and Pmel-1 cells. Fig. S5 shows the effects of IL-1β administration on *Il2*, *Il15*, and *Il15ra* mRNA levels, frequency of IL-2^+^ cells, CD25 and CD122 expression in OT-I cells, frequency and CD25 expression of T reg cells, frequency of FSC^int^SSC^int^ population and CD11b^+^Gr-1^+^ subpopulation, population kinetics of the splenic immune cell types, and the IL-15Rα expression in different immune cell types. Table S1 shows the RNA-seq data analysis for Fig. 2 A.

## Supplementary Material

Supplemental Materials (PDF)

Table S1 (Excel file)
