# Supplementary material for: Host conditioning with IL-1β improves the antitumor function of adoptively transferred T cells
Source: J Exp Med. 2019 Aug 12;216(11):2619–34. doi: 10.1084/jem.20181218 (PMC6829590; doi:10.1084/jem.20181218)
Supplement: Supplemental Materials (PDF) [file JEM_20181218_sm.pdf]

Supplemental material

Lee et al., https://doi.org/10.1084/jem.20181218

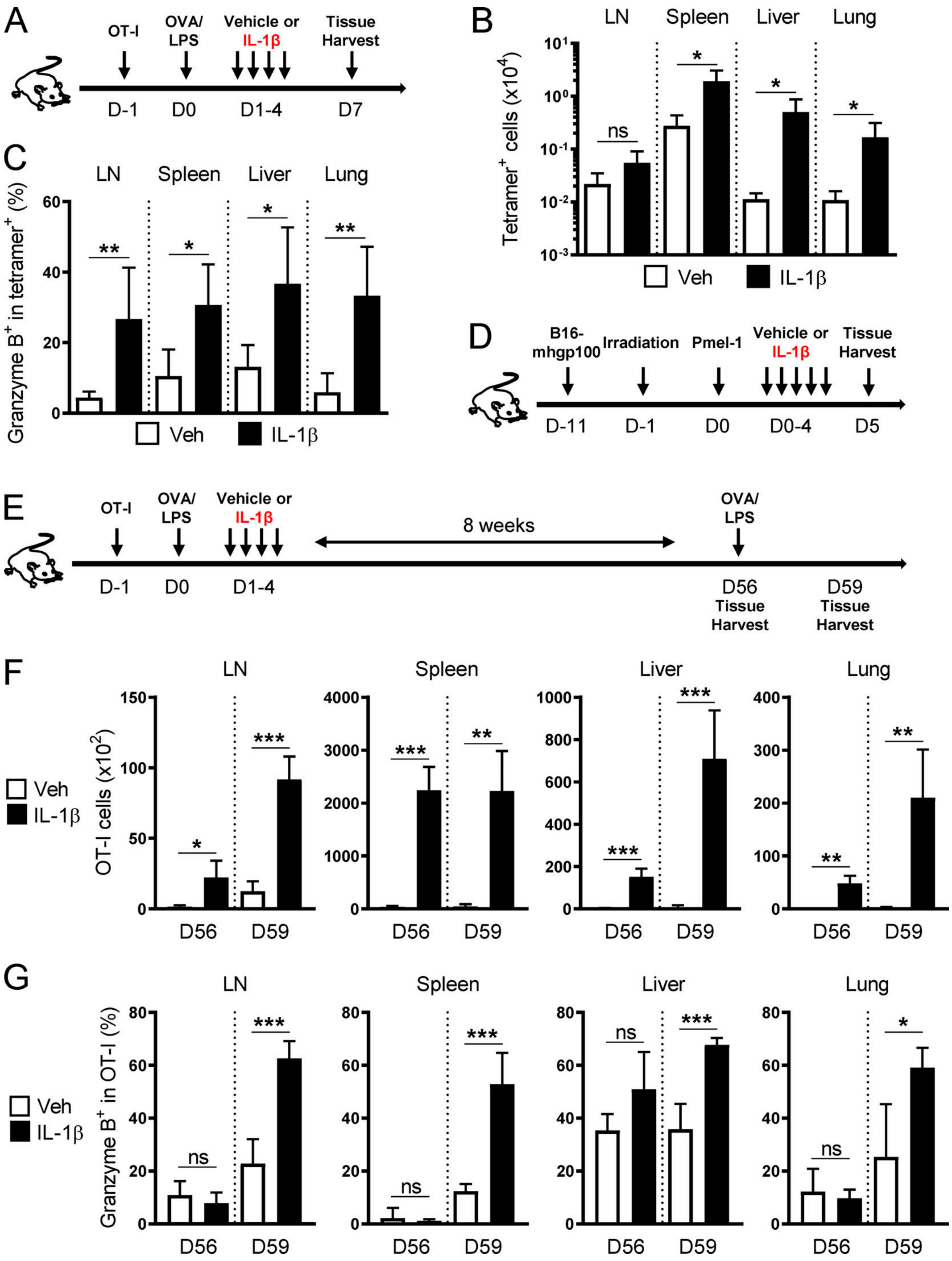

Figure S1. **Administration of IL-1β enhances primary and recall CD8⁺ T cell responses. (A)** A schematic illustrating the generation of a primary OT-I response by OVA/LPS. Naive OT-I cells ($CD45.1^+$) were transferred to a congenic host ($CD45.1^+CD45.2^+$) on day (D) −1, followed by OVA/LPS immunization on day 0 and four daily injections of vehicle or IL-1β on days 1–4. Tissues were harvested on day 7. **(B and C)** Absolute cell number and frequency of SIINFEKL/H2-K^b tetramer⁺ CD8⁺ T cells expressing Gzm B isolated on day 7 from vehicle (white bars)– and IL-1β (black bars)–treated mice as described in A (*n* = 5). **(D)** A schematic illustrating the treatment of B16-mhgp100–bearing mice with the adoptive transfer of Pmel-1 cells. Mice bearing 10-d-old B16-mhgp100 tumors received total body irradiation on day −1, followed by the infusion of naive Pmel-1 cells on day 0 and five daily injections of vehicle or IL-1β on days 0–4. Mice were either euthanized on day 5 for tissue harvesting or kept for ≤60 d for tumor measurements. **(E)** A schematic illustrating the generation of a recall OT-I response by OVA/LPS. Naive OT-I cells were transferred to a congenic host on day −1, followed by OVA/LPS immunization on day 0 and four daily injections of vehicle or IL-1β on days 1–4. An OVA/LPS boost was given on D56, and the tissues were harvested before (D56) and three days after (D59) the boost. **(F and G)** Absolute cell number and frequency of OT-I cells expressing Gzm B isolated on days 56 and 59 from vehicle (white bars)– and IL-1β (black bars)–treated mice as described in E (*n* = 4). Data are representative of two independent experiments (*, $P < 0.05$; **, $P < 0.01$; ***, $P < 0.001$; ns, not significant; error bars, SD).

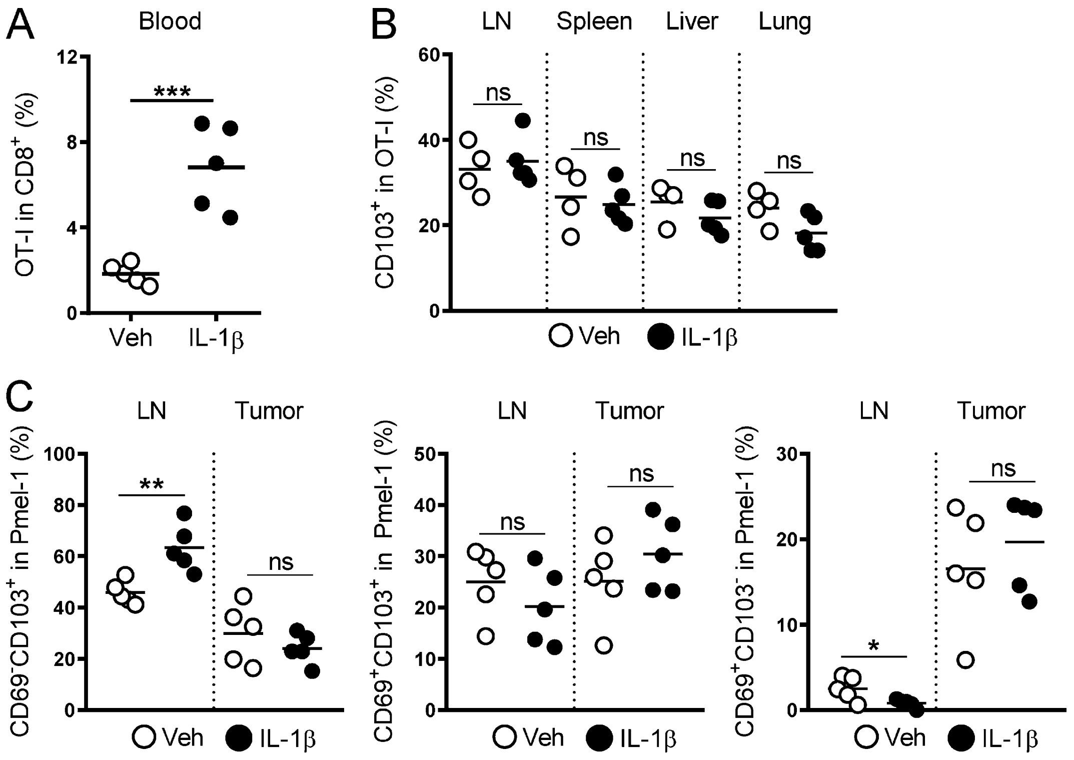

Figure S2. **Administration of IL-1β does not facilitate the acquisition of a Trm phenotype. (A)** Frequency of OT-I cells on day 6 in the blood from vehicle (white circles)– and IL-1β (black circles)–treated mice (n = 5). **(B)** Frequency of OT-I cells expressing CD103 isolated on day 6 from vehicle (white circles)– and IL-1β (black circles)–treated mice (n = 4 or 5). **(C)** Frequency of Pmel-1 cells expressing CD103 alone, CD69 and CD103, and CD69 alone isolated on day 5 from vehicle (white circles)– and IL-1β (black circles)–treated tumor-bearing mice (n = 5). Data are representative of two independent experiments (*, P < 0.05; **, P < 0.01; ***, P < 0.001; ns, not significant).

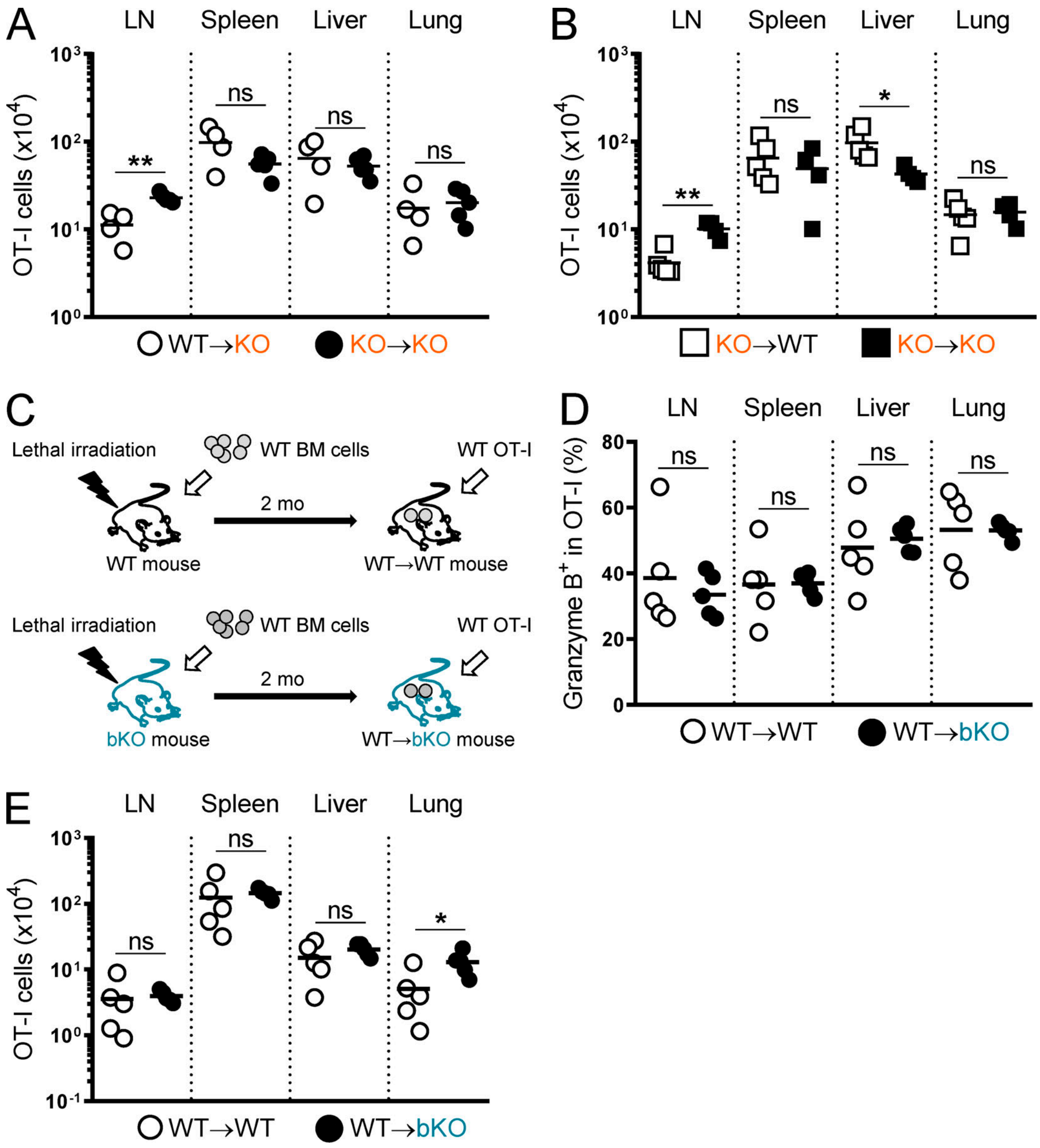

Figure S3. **Differential role of radio-sensitive and radio-resistant host cells in mediating IL-1β enhancement of CD8⁺ T cell responses. (A)** Absolute number of OT-I cells isolated on day 6 from WT→KO (white circles) and KO→KO (black circles) mice treated with OVA/LPS on day 0 and IL-1β on days 1–4 as described in Fig. 6 C (*n* = 4 or 5). **(B)** Absolute number of OT-I cells isolated on day 6 from KO→WT (white squares) and KO→KO (black squares) mice treated with OVA/LPS on day 0 and IL-1β on days 1–4 as described in Fig. 6 E (*n* = 5 or 4). **(C)** A schematic illustrating the generation of bone marrow chimeric mice. WT→WT and WT→β2M KO (bKO) mice were generated by reconstituting lethally irradiated WT or $B2m^{-/-}$ (bKO) mice with WT bone marrow cells, respectively. 2 mo later, WT OT-I cells were transferred to the bone marrow chimeric hosts, followed by OVA/LPS and IL-1β treatments (*n* = 5). **(D and E)** Frequency of OT-I cells expressing Gzm B and absolute OT-I cell number isolated on day 6 from WT→WT (white circles) and WT→bKO (black circles) mice treated with OVA/LPS on day 0 and IL-1β on days 1–4 as described in C. Data are representative of two independent experiments (*, $P < 0.05$; **, $P < 0.01$; ns, not significant).

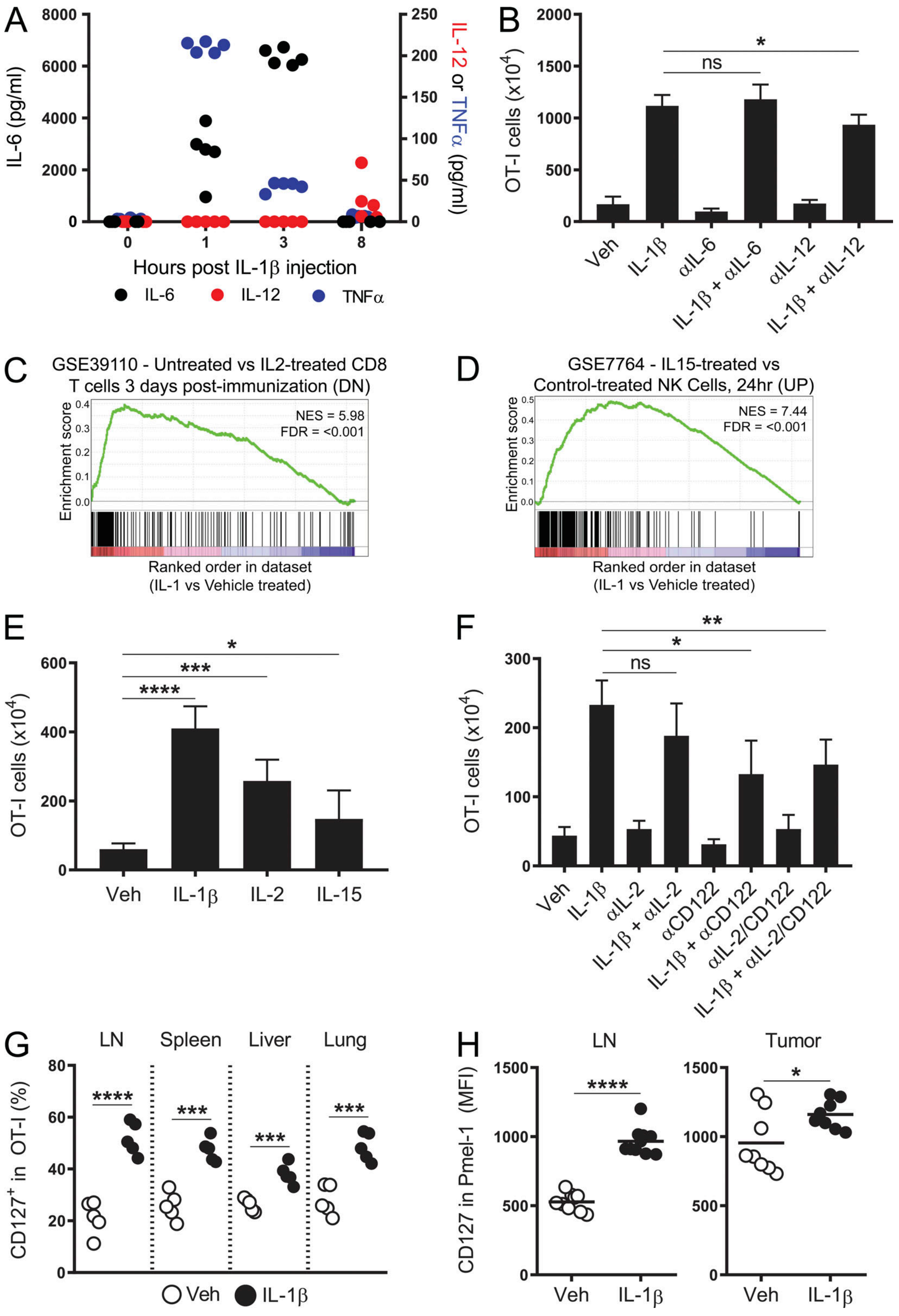

Figure S4. **Role of the cytokine milieu in mediating IL-1β enhancements of CD8+ T cell responses. (A)** Blood samples were collected 0, 1, 3, and 8 h after a single s.c. injection of IL-1β (2 µg). Serum levels of IL-6 (black circles), IL-12p70 (red circles), and TNFα (blue circles) were measured by ELISA (n = 5). **(B)** Absolute number of OT-I cells isolated on day 6 from the spleen of mice injected with OVA/LPS on day 0 and indicated treatments on days 1–4 (n = 5). **(C and D)** GSEA plots showing the enrichment of IL-2- and IL-15-induced genes in IL-1β– versus vehicle-exposed OT-I cells as described in Fig. 2 A. NES, normalized enrichment score; FDR, false discovery rate. **(E and F)** Absolute number of OT-I cells isolated on day 6 from the spleen of mice injected with OVA/LPS on day 0 and indicated treatments on days 1–4 (n = 5). **(G)** Frequency of OT-I cells expressing IL-7Rα (CD127) isolated on day 6 from mice treated with OVA/LPS on day 0 and vehicle or IL-1β on days 1–4 (n = 5). **(H)** Mean fluorescence intensity (MFI) of CD127 expression in Pmel-1 cells isolated on day 5 from B16-mhgp100–bearing mice treated with vehicle or IL-1β on days 0–4 (n = 8). Data are representative of two (A, B, E, and H) or three (F and G) independent experiments (*, $P < 0.05$; **, $P < 0.01$; ***, $P < 0.001$; ****, $P < 0.0001$; ns, not significant; error bars, SD).

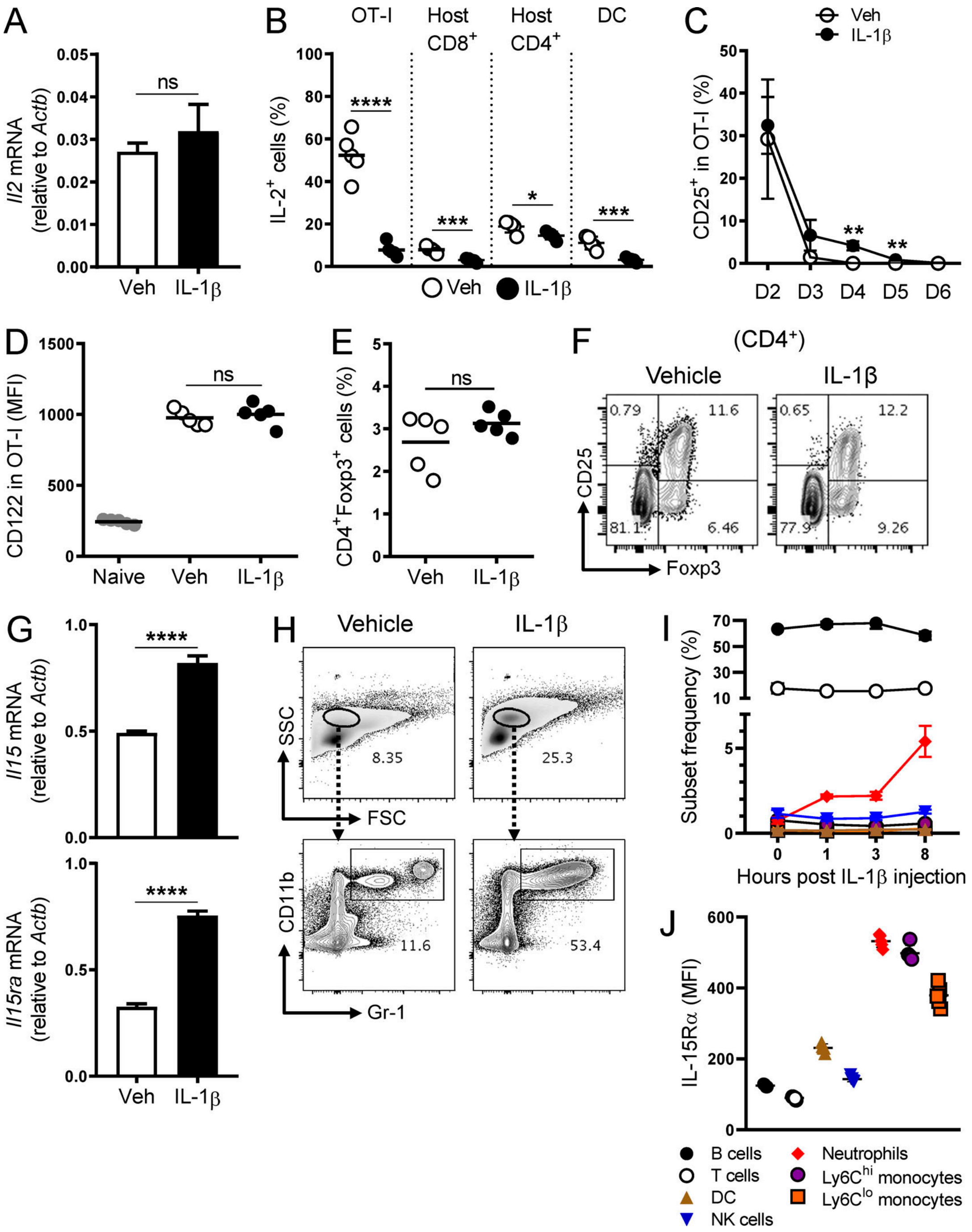

Figure S5. **Impact of IL-1β on the availability of IL-2 and IL-15 to CD8$^+$ T cells. (A)** Levels of the *Il2* mRNA in total splenocytes from vehicle (white bars)– and IL-1β (black bars)–treated mice (*n* = 5). **(B)** Frequency of the indicated cell populations expressing IL-2 in the spleen on day 6 from vehicle (white circles)– and IL-1β (black circles)–treated mice (*n* = 5). OT-I: B220$^-$CD3$^+$CD8$^+$Vα2$^+$CD45.1$^+$CD45.2$^-$, host CD8$^+$: B220$^-$CD3$^+$CD8$^+$CD45.1$^+$CD45.2$^+$, host CD4$^+$: B220$^-$CD3$^+$CD4$^+$CD45.1$^+$CD45.2$^+$, dendritic cell: B220$^-$CD3$^-$CD11c$^+$NK1.1$^-$. **(C)** Frequency of OT-I cells expressing CD25 isolated on days 2–6 from the draining LNs of vehicle (white circles)– and IL-1β (black circles)–treated mice (*n* = 3). **(D)** IL-2/15Rβ (CD122) MFI in OT-I cells isolated on day 6 from the spleen of vehicle (white circles)– and IL-1β (black circles)–treated mice. Host naive CD8$^+$ T cells (naive; CD8$^+$CD44^{lo} gated) were used as a negative control (*n* = 5). **(E)** Frequency of CD4$^+$Foxp3$^+$ cells in total splenocytes isolated on day 6 from vehicle (white circles)– and IL-1β (black circles)–treated mice (*n* = 5). **(F)** Representative contour plots showing Foxp3 and CD25 expression in CD4$^+$ T cells as described in E. **(G)** Levels of the *Il15* and *Il15ra* mRNA in total splenocytes from vehicle (white bars)– and IL-1β (black bars)–treated mice (*n* = 5). **(H)** Representative contour plots showing the frequency of the FSC^{int}SSC^{int} population in the spleen on day 6 from vehicle- and IL-1β–treated mice. The frequency of CD11b$^+$Gr-1$^+$ cells within the FSC^{int}SSC^{int} population is shown below. **(I)** Population kinetics of the indicated cell types in the spleen upon a single injection of IL-1β (2 µg). B cells: B220$^+$CD3$^-$, T cells: B220$^-$CD3$^+$, DC: B220$^-$CD3$^-$CD11c$^+$NK1.1$^-$, NK cells: B220$^-$CD3$^-$NK1.1$^+$, neutrophils: B220$^-$CD3$^-$CD11c$^-$NK1.1$^-$CD11b$^+$Ly6G$^+$Ly6C^{int}, Ly6C^{hi} monocytes: B220$^-$CD3$^-$CD11c$^-$NK1.1$^-$CD11b$^+$Ly6G$^-$Ly6C^{hi}, Ly6C^{lo} monocytes: B220$^-$CD3$^-$CD11c$^-$NK1.1$^-$CD11b$^+$Ly6G$^-$Ly6C^{lo} (*n* = 5). **(J)** MFI of IL-15Rα expression in the indicated cell types in the spleen (*n* = 5). Data are representative of two (A–D and G–J) or four (E and F) independent experiments (*, P < 0.05; **, P < 0.01; ***, P < 0.001; ****, P < 0.0001; ns, not significant; error bars, SD).

Table S1 is provided online as a separate Excel file and shows RNA-seq analysis of the global transcriptome profile of IL-1β– versus vehicle-exposed OT-I cells in vivo.
